# 

**Published:** 1875-07

**Authors:** 


### TABLE OF CONTENTS.

ORIGINAL COMMUNICATIONS.

Old Eyes and Spectacles. By Swan M. Burnett, M.D.... 385
Five Cases of Operation by the Bloodless Method. By Lewis Balch, M.D... 391
Clinical Notes. By F. K. Bailey, M.D.... 394
A case of Albuminuric Eclampsia. By G. L. Ullman, M.D.... 397
Concussion of the Brain. By J. D. Rush, M.D.... 401

SELECTIONS.

A Contribution to the Treatment of Epistaxis. By Beverly Robinson, M.D... 402
The Contagion of Puerperal Fever.... 406
Sulphide of Calcium in Scrofulous Abscesses and Boils. By T. Curtis Smith, M.D.... 409
Two New Differential Signs in Dislocations of the Shoulder. By Prof. H. Hamilton, M.D.... 412
Another Method of Perforating the Drum Membrane. By J. Simrock, M.D. 415
On Hydrocele of the Neck—A Clinical Note. By Sampson Gamgee, F.R.S.E. 417

EXTRACTS AND GLEANINGS.

Indications for Thoracentesis—419. Diphtheria—420. Hydrochloral by the Rectum in the Vomiting of Pregnancy—421. Bismuth in Skin Disease—422. Nux Vomica in Nervous Diseases—423. Burns and Scalds; An Antidote to Chloroform—424. External Use of Tincture of Iron in Erysipelas; Diarrhœa Mixture—325. Anomalous Digestion; Chloral Suppositories—426. Treatment of Sudden Intestinal Occlusion by Opium; Salicylic Acid as a Disinfectant—427. Puncture of the Bladder for the Retention of Urine; Pulsatilla in Amenorrhœa—428. Copaiba in Dropsy; Bicarbonate of Soda in the Treatment of Toothache—429. Treatment of Torticollis; Fucus Poultices—430. Hydrastin in Gonorrhœa; Diagnosis of Disease of the Liver—431. Nitrogeneous Diet in Rheumatism; Bromide of Ammonia in Catamenial Excesses—432. The Treatment of Fractured Patella—433. Ergot in Cystic Paralysis; Ingrafting of Tendons; Anti-Gastralgic Drops—434. Digitalis in Typhoid Fever; Ointment for Sycosis; Treatment of Delirium Tremens—435. The Remedial Use of Sea-Water as a Beverage; Diphtheria—436. Idioform in Chronic Suppuration of the Middle Ear; The Cyanides in Acute Articular Rheumatism—437. The Sulphuret of Carbon in Chronic and Atonic Ulcers; Guarana in Chronic Rheumatism—438. On the Use of Chocolate in Chronic Intestinal Catarrh; Wood-Sores in Epithelioma—439. Treatment of Chorea by Arsenic in Large Doses; Salicylic Acid in Extensive Burn; Bromide of Potassium in Obstinate Vomiting; Magendie's Solution of Morphine—440. Solubility of Salicylic Acid; Obstinate Vomiting in Pregnancy Treated by Dilatation of the Os Uteri; Characteristically Spanish—441. Retained Placenta; Quinia in Parturition; Salicylic Acid—442. New Method of Administering Raw Meat; Chorea; Strychnia as an Anti-Emetic; Incontinence of Urine—443. Russian Cure for Drunkenness; Ergotin in Fibroid of the Uterus; Sciatica; A Convenient Local Anodyne—444.

EDITORIAL.

Medical Colleges.... 445

To our Readers.... 447

